# Healthcare vulnerability disparities in pancreatic cancer treatment and mortality using the Korean National Sample Cohort: a retrospective cohort study

**DOI:** 10.1186/s12885-022-10027-2

**Published:** 2022-08-27

**Authors:** Sung Hoon Jeong, Hyeon Ji Lee, Choa Yun, Il Yun, Yun Hwa Jung, Soo Young Kim, Hee Seung Lee, Sung-In Jang

**Affiliations:** 1 Department of Public Health, Graduate School, Yonsei University, Seoul, Republic of Korea; 2 Institute of Health Services Research, Yonsei University, 50 Yonsei-ro, Seodaemun-gu, 03722 Seoul, Republic of Korea; 3 Department of Biostatistics & Computing, College of Medicine, Yonsei University, Seoul, Republic of Korea; 4 Division of Gastroenterology, Department of Internal Medicine, Yonsei University College of Medicine, Seoul, Republic of Korea; 5 Department of Preventive Medicine, Yonsei University College of Medicine, 50 Yonsei-ro, Seodaemun-gu, Seoul, 03722 Republic of Korea

**Keywords:** Regional disparities, Pancreatic cancer, Epidemiology, Pancreatic cancer treatment, Healthcare vulnerability

## Abstract

**Background:**

The gap in treatment and health outcomes after diagnosis of pancreatic cancer is a major public health concern. We aimed to investigate the differences in the health outcomes and treatment of pancreatic cancer patients in healthcare vulnerable and non-vulnerable areas.

**Methods:**

This retrospective cohort study evaluated data from the Korea National Health Insurance Corporation-National Sample Cohort from 2002 to 2019. The position value for relative comparison index was used to define healthcare vulnerable areas. Cox proportional hazard regression was used to estimate the risk of mortality in pancreatic cancer patients according to healthcare vulnerable areas, and multiple logistic regression was used to estimate the difference in treatment.

**Results:**

Among 1,975 patients, 279 (14.1%) and 1,696 (85.9%) lived in the healthcare vulnerable and non-vulnerable areas, respectively. Compared with the non-vulnerable area, pancreatic cancer patients in the vulnerable area had a higher risk of death at 3 months (hazard ratio [HR]: 1.33, 95% confidence interval [CI] = 1.06–1.67) and 6 months (HR: 1.23, 95% CI = 1.03–1.48). In addition, patients with pancreatic cancer in the vulnerable area were less likely to receive treatment than patients in the non-vulnerable area (odds ratio [OR]: 0.70, 95% CI = 0.52–0.94). This trend was further emphasized for chemotherapy (OR: 0.68, 95% CI = 0.48–0.95).

**Conclusion:**

Patients with pancreatic cancer belonging to medically disadvantaged areas receive less treatment and have a higher risk of death. This may be a result of the late diagnosis of pancreatic cancer among these patients.

**Supplementary Information:**

The online version contains supplementary material available at 10.1186/s12885-022-10027-2.

## Introduction

Worldwide, pancreatic cancer is the 12^th^ most common malignancy and the 7^th^ leading cause of cancer mortality [1]. The prognosis for pancreatic cancer is poor, with long-term survival rates of 9% despite various advances in combination therapy [2]. Surgical resection remains the only potential cure for pancreatic cancer. However, since most tumors are locally advanced or metastatic at the time of diagnosis, only 15% to 20% of patients are eligible for resection [3]. Several studies have reported that the 5-year survival rate of surgical patients is as high as 30% [4–6]. However, surgical methods such as removal of most pancreatic infiltrates are feasible when pancreatic cancer is diagnosed at an early stage. Chemotherapy is preferred when the diagnosis is at later stages [7, 8]. Surgery and chemotherapy may not be feasible, especially if pancreatic cancer is discovered in advanced stages [7, 8]. Therefore, staging pancreatic cancer at the time of diagnosis is particularly important as it has a significant impact on treatment options and survival [9, 10]. Nevertheless, the main concern of the geographically disadvantaged population that has a poor survival rate is the disparities in the diagnosis stage of cancer [11].

Research on disparities in pancreatic cancer outcomes is predominantly reported from western countries, such as the United States, and focused on disparities by race and ethnicity or type of health insurance [12, 13]. Significant interest in racial and ethnic imbalances is key in improving outcomes, but patients living in healthcare vulnerable areas, facing socioeconomic problems and travel costs are often overlooked, which may affect both treatment options and pancreatic cancer outcomes [14]. The presence or absence of residence in a healthcare vulnerable area can lead to differences in the stage at which the cancer is diagnosed and differences in treatment availability [15]. Where there are differences in treatment availability, apart from racial disparity, economic status, and insurance coverage [16–18], the role of regional disparity variables on pancreatic cancer outcomes remains unclear. Thus, there is limited evidence of regional disparity in early diagnosis and post-diagnosis treatment and health outcomes among patients diagnosed with pancreatic cancer.

In addition, previous studies tend to focus on dichotomizing regional disparities into rural and urban areas [14]. However, patients living in remote areas may have greater difficulties in accessing timely care. Therefore, it is necessary to comprehensively consider the level of healthcare between regions and specifically investigate whether to treat pancreatic cancer after diagnosis and health outcomes.

To define the level of healthcare between regions, we used the position value for relative comparison (PARC) index, a measure that can relatively evaluate the level of health care between regions. The PARC index has been widely used in linear studies to diagnose the level of health care by region [15–17]. Therefore, this study aimed to classify healthcare vulnerable and non-vulnerable areas using the PARC index through Korean nationwide claims data and investigate the difference between treatment and health outcomes of pancreatic cancer patients in healthcare vulnerable and non-vulnerable areas. We investigated the following two hypotheses: 1) mortality from all causes will be higher in vulnerable areas than in non-vulnerable areas; patients who have undergone surgery and chemotherapy will have higher mortality from all causes than those who did not; 2) compared to non-vulnerable areas, there will be fewer surgeries and chemotherapy that can indirectly examine early diagnosis in vulnerable areas.

## Materials and methods

All data are available in the database of the Korean National Health Insurance Sharing Service (https://nhiss.nhis.or.kr) and can be accessed upon reasonable request. This study was reviewed and approved by the International Review Board of Yonsei University’s Health System (IRB number: Y-2020–0031) and adheres to the tenets of the Declaration of Helsinki. The Korea National Health Insurance Service-National Sample Cohort (NHIS-NSC) data do not contain any identifying information. Due to the retrospective nature of the study, the requirement to obtain informed consent was waived.

### Study population and data

The data analyzed in this study were acquired from the Korean National Health Insurance Service National Sample Cohort (NHIS-NSC) of 2002 and 2019, from the National Health Insurance Service (NHIS). The Korean NHIS provides researchers with all claims data collected by the corporation for academic research and policymaking. The data for this study were collected from insurance claims that included demographic information, diagnosis, medications, costs, date of visit, and date of death, if applicable. As of 2002, out of 47,851,928 people, excluding foreigners, 46,605,433 participants were selected for the sample cohort. From the full NHIS databas, the representative sample cohort consisted of 1,025,340 people, which were randomly stratified from 2.2% of the total population of Korea [18]. Follow-up data were available through 2019 and included information on medical claims filed between 2002 and 2019.

During the study period, a total of 3,454 patients were newly diagnosed with pancreatic cancer, according to the International Classification of Diseases (ICD-10 code: C25). First, patients diagnosed with pancreatic cancer between 2002 and 2003 were excluded to ensure a pancreatic cancer-free period of at least 2 years(*n* = 227). This eliminated the effects of pancreatic cancer that might have occurred prior to the cohort observation period. Second, to increase the accuracy of new cancer diagnoses, patients without the V027, V193, and V194 codes, which are domestic cancer-only self-pay codes, were excluded (*n* = 1,146) [19]. Finally, participants with missing information on covariates such as age, sex, social security status, disability, and household income level were excluded, including those under 19 years of age at the time of pancreatic cancer diagnosis (*n* = 106). After this exclusion, 1,975 patients with the first diagnosis of pancreatic cancer were included in the study.

The last date of follow-up was defined as the date of death or December 31, 2019, whichever occurred first. Index date (time 0) was defined as the date of the first pancreatic cancer diagnosis that met the eligibility criteria for a patient with pancreatic cancer (either outpatient care or inpatient care; ICD-10: C25).

### Study variables and covariates

In this study, the variable of interest was the healthcare vulnerable region. The PARC index, was used to diagnose the level of healthcare by region in Korea [15–17]. PARC is an objective indicator that can identify relative locations compared to other regions concerning medical demand, supply, access, use, and health conditions in each region. The PARC value is between -1 and 1, and when compared with the average value of the entire region, the value is considered best when it is 1, 0 for the average, and -1 for the worst [15]. Thus, a PARC value closer to -1 is associated with a lower than average level of healthcare care in the area, whereas that closer to 1 is associated with higher levels of healthcare care in the area [15]. In this study, when the PARC value was less than -0.33, it was classified as a healthcare vulnerable region.

The primary health outcome variable in this study was all-cause mortality. The primary health outcome variable in this study was all-cause mortality. Though most previous studies on pancreatic cancer report 5-year survival rates [20, 21], our study measured mortality at 3 months, 6 months, and 1-year as pancreatic cancer is characterized by an average survival period of 3 to 6 months and a poor prognosis [22, 23]. In NHIS, each patient’s unique de-identification number was linked to the mortality information of the National Statistical Office [18]. The time from index date to death date was used to define survival time. The secondary outcome variable was treatment according to healthcare vulnerability. As treatment choice would indirectly indicate the time taken for initial diagnosis, the treatment was divided into surgery and chemotherapy. Based on available literature and expert opinions, 8 therapies in cases of pancreatic cancer surgery were included. Gemcitabine and 5-fluorouracil drugs were included (Supplementary Table 1) [24, 25].

Possible confounding factors in this study were variables that could affect mortality and treatment availability in patients with pancreatic cancer. This included age, sex, social security status, disability, household income level, Charlson Comorbidity Index (CCI), and year of diagnosis of pancreatic cancer. The CCI score is an index for evaluating a participant’s comorbidities that may alter the risk of death, for use in longitudinal studies. The score was calculated by weighting 1 to 6 points for 19 comorbidities. The categories included in the CCI score are myocardial, vascular, lung, endocrine, kidney, gastrointestinal, cancer/immune, and neurological comorbidities [26]. Participants’ CCI scores were calculated using ICD-10 codes for each comorbidity [27]. Participants were divided into the following three groups according to their CCI scores: 0, 1–2, and ≥ 3. The age group was classified considering pancreatic cancer’s high incidence after the age of 50 years (< 50 years, 50–60 years, 60–70 years, 70–80 years, or > 80 years) [28]. Social security status was classified according to the health insurance premiums of the employee insured or self-employed insured categories, according to the standards of Korea’s NHIS. Medical assistance beneficiaries were persons with disabilities with incomes below government-set poverty standards or people who are eligible for free inpatient and outpatient care by the government. Household income level was classified into three categories according to household-level insurance premiums, namely low, mid, and high. Disability was classified into two categories (yes and no) depending on whether the rating was determined.

### Statistical analyses

At baseline (time point 0), the frequency and percentage of each categorical variable were investigated, and chi‐squared tests were performed to investigate the distribution of mortality according to each variable. Furthermore, we investigated at the distribution of mortality from pancreatic cancer (ICD-10 code: C25). A Cox proportional hazards model investigated the association between healthcare vulnerability and treatment presence and all-cause mortality in patients with the first diagnosis of pancreatic cancer. All Cox proportional hazards models were fully adjusted for the covariates presented in Table 1. The results were presented as hazard ratios (HRs) with 95% confidence intervals (CIs). We also performed stratified analyses using the data on pancreatic cancer treatment, sex, and household income to investigate the association between healthcare vulnerability and mortality in pancreatic cancer patients. Further, to examine whether pancreatitis treatment was according to healthcare vulnerability, a multiple logistic regression analysis was performed after adjusting all the covariates presented in Table 1. Furthermore, multinomial logistic regression analysis was performed to examine whether treatment was performed according to healthcare vulnerability by dividing it into surgery and chemotherapy. The results were reported as an odds ratio (OR) with a 95% CI.

**Table 1:** General characteristics of the study population according to the diagnosis of pancreatic cancer

Variables	Total		3-month mortality				*P*-value	6-month mortality				*P*-value	1-year Mortality				*P*-value
			No		Yes			No		Yes			No		Yes		
Total	1,975	(100.0)	1,498	(75.8)	477	(24.2)		1,191	(60.3)	784	(39.7)		836	(42.3)	1,139	(57.7)	
**Healthcare Vulnerability**							< .0001					0.0003					0.0180
Vulnerable region	279	(14.1)	184	(65.9)	95	(34.1)		141	(50.5)	138	(49.5)		100	(35.8)	179	(64.2)	
Non-vulnerable region	1,696	(85.9)	1,314	(77.5)	382	(22.5)		1,050	(61.9)	646	(38.1)		736	(43.4)	960	(56.6)	
**Pancreatic Cancer Treatment**							< .0001					< .0001					< .0001
Yes	860	(43.5)	795	(92.4)	65	(7.6)		676	(78.6)	184	(21.4)		455	(52.9)	405	(47.1)	
No	1,115	(56.5)	703	(63.0)	412	(37.0)		515	(46.2)	600	(53.8)		381	(34.2)	734	(65.8)	
**Sex**							0.3964					0.7728					0.5420
Male	1,093	(55.3)	821	(75.1)	272	(24.9)		656	(60.0)	437	(40.0)		456	(41.7)	637	(58.3)	
Female	882	(44.7)	677	(76.8)	205	(23.2)		535	(60.7)	347	(39.3)		380	(43.1)	502	(56.9)	
**Age**							< .0001					< .0001					< .0001
< 50	173	(8.8)	157	(90.8)	16	(9.2)		138	(79.8)	35	(20.2)		122	(70.5)	51	(29.5)	
50–60	311	(15.7)	275	(88.4)	36	(11.6)		234	(75.2)	77	(24.8)		163	(52.4)	148	(47.6)	
60–70	503	(25.5)	415	(82.5)	88	(17.5)		350	(69.6)	153	(30.4)		251	(49.9)	252	(50.1)	
70–80	598	(30.3)	431	(72.1)	167	(27.9)		320	(53.5)	278	(46.5)		217	(36.3)	381	(63.7)	
> 80	390	(19.7)	220	(56.4)	170	(43.6)		149	(38.2)	241	(61.8)		83	(21.3)	307	(78.7)	
**Household income**							0.7841					0.3680					0.2443
Low	437	(22.1)	326	(74.6)	111	(25.4)		251	(57.4)	186	(42.6)		171	(39.1)	266	(60.9)	
Mid-low	621	(31.4)	474	(76.3)	147	(23.7)		377	(60.7)	244	(39.3)		275	(44.3)	346	(55.7)	
Mid-high	917	(46.4)	698	(76.1)	219	(23.9)		563	(61.4)	354	(38.6)		390	(42.5)	527	(57.5)	
**Medical Insurance**							0.8633					0.5623					0.2294
Insurance Coverage (Regional)	639	(32.4)	480	(75.1)	159	(24.9)		381	(59.6)	258	(40.4)		262	(41.0)	377	(59.0)	
Insurance Coverage (corporate)	1,279	(64.8)	975	(76.2)	304	(23.8)		779	(60.9)	500	(39.1)		555	(43.4)	724	(56.6)	
Medical Aid	57	(2.9)	43	(75.4)	14	(24.6)		31	(54.4)	26	(45.6)		19	(33.3)	38	(66.7)	
**Disorder**							0.0355					0.0168					0.0339
No	1,828	(92.6)	1,397	(76.4)	431	(23.6)		1,116	(61.1)	712	(38.9)		786	(43.0)	1,042	(57.0)	
Yes	147	(7.4)	101	(68.7)	46	(31.3)		75	(51.0)	72	(49.0)		50	(34.0)	97	(66.0)	
**CCI**							0.0003					0.0269					0.8036
0	353	(17.9)	262	(74.2)	91	(25.8)		211	(59.8)	142	(40.2)		145	(41.1)	208	(58.9)	
1–2	885	(44.8)	708	(80.0)	177	(20.0)		561	(63.4)	324	(36.6)		381	(43.1)	504	(56.9)	
≥ 3	737	(37.3)	528	(71.6)	209	(28.4)		419	(56.9)	318	(43.1)		310	(42.1)	427	(57.9)	

*CCI* Charlson Comorbidity Index

All statistical analyses were performed using SAS software, version 9.4 (SAS Institute Inc., Cary, NC, USA). Statistical significance was set at *p* < 0.05.

## Result

Table 1 describes general characteristics and mortality at baseline. Of the 1,975 patients diagnosed with pancreatic cancer for the first time; 279 (14.1%) and 1,696 (85.9%) lived in the healthcare vulnerable non-vulnerable regions, respectively. Furthermore, 860 of 1,975 (43.5%) received treatment such as surgery or chemotherapy, and 1,115 (56.5%) did not receive treatment. Moreover, of the 860 treated patients, 313 (15.8%) underwent surgery and 547 (27.7%) underwent chemotherapy (Supplementary Table 2). All-cause mortality after initial diagnosis of pancreatic cancer was noted in 477 (24.2%), 784 (39.7%), and 1,139 (57.7%) at 3 months, 6 months, and 1-year, respectively. Healthcare vulnerability and treatment status of the first diagnosed pancreatic cancer cases showed a significant difference in the mortality rate at 3 months, 6 months, and 1-year. Furthermore, the pancreatic cancer specific mortality rates in patients with pancreatic cancer diagnosed for the first time and were 382 (19.3%), 625 (31.6%), and 895 (45.3%) at 3 months, 6 months, and 1-year, respectively. However, there was a statistically significant difference between healthcare vulnerability and pancreatic cancer specific mortality only at 3 and 6 months (Supplementary Table 3).

Table 2 shows the results of survival analysis using the Cox proportional hazards model, which investigated the association between the healthcare vulnerable area and treatment status of pancreatic cancer patients and the all-cause mortality. Pancreatic cancer patients in vulnerable areas had higher mortality at 3 months (HR = 1.33, 95% CI = 1.06–1.67), 6 months (HR = 1.23, 95% CI = 1.03–1.48), and 1-year (HR = 1.13, 95% CI = 0.96–1.33) compared to patients in non-vulnerable areas. However, 1-year all-cause mortality was not statistically significant. Moreover, the group that did not receive treatment for pancreatic cancer had higher mortality at 3 months (HR = 4.85, 95% CI = 3.69–6.37), 6 months (HR = 2.91, 95% CI = 2.44–3.46), and 1-year (HR = 1.78, 95% CI = 1.56–2.03) compared to the group that received treatment for pancreatic cancer, and the differences were statistically significant.

**Table 2:** Cox proportional hazards regression analysis results for the association between healthcare vulnerable areas and all-cause mortality

Variable	3-month mortality				6-month mortality				1-year Mortality			
	**Adjusted** **HR**	**95% CI**			**Adjusted** **HR**	**95% CI**			**Adjusted** **HR**	**95% CI**		
**Healthcare Vulnerability**												
Vulnerable region	1.33	(1.06	-	1.67)	1.23	(1.03	-	1.48)	1.13	(0.96	-	1.33)
Non-vulnerable region	1.00				1.00				1.00			
**Pancreatic Cancer Treatment**												
Yes	1.00				1.00				1.00			
No	4.85	(3.69	-	6.37)	2.91	(2.44	-	3.46)	1.78	(1.56	-	2.03)
**Sex**												
Male	1.00				1.00				1.00			
Female	0.80	(0.66	-	0.96)	0.84	(0.72	-	0.97)	0.85	(0.75	-	0.96)
**Age**												
< 50	1.00				1.00				1.00			
50–60	1.40	(0.78	-	2.53)	1.40	(0.94	-	2.10)	1.90	(1.38	-	2.61)
60–70	2.26	(1.32	-	3.86)	1.87	(1.29	-	2.70)	2.18	(1.61	-	2.95)
70–80	3.25	(1.93	-	5.45)	2.88	(2.02	-	4.12)	3.17	(2.36	-	4.27)
≥ 80	4.09	(2.44	-	6.88)	3.51	(2.45	-	5.04)	4.29	(3.17	-	5.80)
**Household income**												
Low	1.00				1.00				1.00			
Mid-low	0.88	(0.68	-	1.14)	0.88	(0.72	-	1.07)	0.88	(0.74	-	1.04)
Mid-high	0.81	(0.63	-	1.02)	0.79	(0.65	-	0.95)	0.81	(0.69	-	0.94)
**Medical Insurance**												
Insurance Coverage(Regional)	1.00				1.00				1.00			
Insurance Coverage(corporate)	0.96	(0.79	-	1.16)	0.95	(0.82	-	1.10)	0.91	(0.80	-	1.03)
Medical Aid	0.69	(0.39	-	1.23)	0.78	(0.51	-	1.20)	0.85	(0.60	-	1.21)
**Disorder**												
No	1.00				1.00				1.00			
Yes	1.18	(0.93	-	1.51)	1.21	(1.00	-	1.47)	1.21	(1.02	-	1.43)
**CCI**												
0	1.00				1.00				1.00			
1–2	0.69	(0.53	-	0.89)	0.79	(0.65	-	0.97)	0.84	(0.71	-	0.99)
≥ 3	0.78	(0.61	-	1.01)	0.79	(0.65	-	0.97)	0.77	(0.65	-	0.91)

*HR* Hazard ratio, *CI* Confidence interval, *CCI*, Charlson Comorbidity Index

Table 3 shows the results of subgroups analysis stratified by pancreatic cancer treatment, sex, and household income. Using the healthcare non-vulnerable group as a reference, we found that in the healthcare vulnerable group, those who were not treated for pancreatic cancer (3 months, HR = 1.36, 95% CI = 1.07–1.72; 6 months, HR = 1.23, 95% CI = 1.00–1.51) and male (3 months, HR = 1.62, 95% CI = 1.22–2.15; 6 months, HR = 1.54, 95% CI = 1.22–1.94; 1-year, HR = 1.30, 95% CI = 1.06–1.60) had higher mortality. Further, regarding household income, the highest mortality was observed in the low-income group (3 months, HR = 1.64, 95% CI = 1.00–2.70; 1-year, HR = 1.45, 95% CI = 1.04–2.04).

**Table 3:** Subgroup analysis of association between healthcare vulnerable areas and all-cause mortality stratified by independent variables

	**Non-vulnerable** **region**	**Vulnerable region**												
		**3-month mortality**				**6-month mortality**				**1-year Mortality**				
	Adjusted HR	**Adjusted** HR*	95% CI			**Adjusted** HR*	95% CI			**Adjusted** HR*	95% CI			
**Pancreatic Cancer Treatment**														
**Yes**	1.00	1.05	(0.49	-	2.21)		1.20	(0.78	-	1.85)	1.01	(0.74	-	1.38)
**No**	1.00	1.36	(1.07	-	1.72)		1.23	(1.00	-	1.51)	1.17	(0.97	-	1.41)
**Sex**														
Male	1.00	1.62	(1.22	-	2.15)		1.54	(1.22	-	1.94)	1.30	(1.06	-	1.60)
Female	1.00	1.00	(0.68	-	1.47)		0.91	(0.67	-	1.24)	0.94	(0.73	-	1.22)
**Household income**														
Low	1.00	1.64	(1.00	-	2.70)		1.40	(0.93	-	2.09)	1.45	(1.04	-	2.04)
Mid-low	1.00	1.33	(0.88	-	2.01)		1.31	(0.94	-	1.82)	1.13	(0.85	-	1.52)
Mid-high	**1.00**	1.17	(0.82	-	1.67)		1.17	(0.88	-	1.56)	1.07	(0.83	-	1.37)

*HR* Hazard ratio, *CI* Confidence interval

^*^ Adjusted for other covariates

Table 4 shows the results of multiple logistic regression and multinomial logistic regression analyses, which were performed to identify the association between treatment and healthcare vulnerability after adjusting all the variables in Table 1. Pancreatic cancer patients in vulnerable areas were less likely to receive treatment than patients in non-vulnerable areas (OR = 0.70; 95% CI = 0.52–0.94). Furthermore, pancreatic cancer patients in vulnerable areas were less likely to undergo surgery (OR = 0.74; 95% CI = 0.50–1.11) and chemotherapy (OR = 0.68; 95% CI = 0.48–0.95) than patients in non-vulnerable areas; however, this association was statistically significant only for chemotherapy treatment.

**Table 4:** Association between healthcare vulnerable areas of pancreatic cancer patients and pancreatic cancer treatment

Variables	Pancreatic Cancer Treatment				No treatment	Surgery				Chemotherapy			
	**OR** ^a^	**95% CI**				**OR** ^b^	**95% CI**			**OR** ^b^	**95% CI**		
**Healthcare Vulnerability**													
Vulnerable region	0.70	(0.52	-	0.94)		0.74	(0.50	-	1.11)	0.68	(0.48	-	0.95)
Non-vulnerable region	1.00				1.00								
**Sex**													
Male	1.00				1.00								
Female	0.98	(0.80	-	1.19)		1.04	(0.80	-	1.36)	0.94	(0.75	-	1.18)
**Age**													
< 50	1.00				1.00								
50–60	1.39	(0.95	-	2.03)		1.05	(0.64	-	1.72)	1.65	(1.07	-	2.55)
60–70	1.43	(1.00	-	2.04)		1.15	(0.73	-	1.82)	1.64	(1.09	-	2.47)
70–80	0.80	(0.56	-	1.14)		0.61	(0.38	-	0.97)	0.95	(0.63	-	1.43)
≥ 80	0.13	(0.08	-	0.20)		0.14	(0.07	-	0.24)	0.12	(0.07	-	0.21)
**Household income**													
Low	1.00				1.00								
Mid-low	1.06	(0.80	-	1.41)		1.25	(0.85	-	1.83)	0.97	(0.71	-	1.33)
Mid-high	1.21	(0.93	-	1.58)		1.33	(0.92	-	1.92)	1.15	(0.86	-	1.55)
**Medical Insurance**													
Insurance Coverage(Regional)	1.00				1.00								
Insurance Coverage(corporate)	1.17	(0.95	-	1.45)		1.25	(0.94	-	1.67)	1.13	(0.89	-	1.43)
Medical Aid	1.35	(0.71	-	2.58)		1.10	(0.41	-	2.93)	1.49	(0.73	-	3.03)
**Disorder**													
No	1.00				1.00								
Yes	0.71	(0.52	-	0.96)		0.86	(0.57	-	1.29)	0.63	(0.43	-	0.90)
**CCI**													
0	1.00				1.00								
1–2	1.13	(0.86	-	1.48)		1.30	(0.90	-	1.86)	1.04	(0.77	-	1.41)
≥ 3	0.51	(0.39	-	0.68)		0.55	(0.37	-	0.81)	0.49	(0.36	-	0.68)

*OR* Odds ratio, *CI* Confidence interval, *CCI* Charlson Comorbidity Index

^a^ Results of association between healthcare vulnerability and treatment of pancreatic cancer patients using multiple logistic regression

^b^ Results of association between healthcare vulnerability, surgery, and chemotherapy in pancreatic cancer patients using multinomial logistic regressions

## Discussion

Pancreatic cancer remains the leading cause of cancer-related deaths. However, in pancreatic cancer, the relative burden continues to increase, with limited progress in the prevention and treatment methods. Although pancreatic cancer can affect any patient population, regardless of patient demographics, certain patient groups may have higher mortality rates than those of others owing to the disproportionate burden of delayed cancer treatment [29].

Our findings emphasize the difference between the vulnerable areas and non-vulnerable areas in terms of health outcomes, such as pancreatic cancer treatment and mortality. First, we found that the health outcomes, i.e., mortality in pancreatic cancer patients in vulnerable areas was higher for 6 months than those of patients in non-vulnerable areas. Furthermore, the mortality rate was high in the group that did not receive treatment after the diagnosis of pancreatic cancer, regardless of the region. Second, compared to patients in the non-vulnerable areas, pancreatic cancer patients in vulnerable areas were less likely to receive treatment related to pancreatic cancer, especially chemotherapy. This indirectly indicates that the diagnosis of pancreatic cancer patients in vulnerable areas is delayed compared to those in non-vulnerable areas. Therefore, our results show that pancreatic cancer patients in healthcare vulnerable areas have a higher mortality rate and are less likely to be diagnosed at an early stage than patients in non-vulnerable areas.

Our study found that the regional differences in pancreatic cancer patients related to mortality or treatment are consistent with the results of previous studies [14, 29–31]. According to previous studies, pancreatic cancer patients in rural areas had a higher mortality rate than that of pancreatic cancer patients in the urban areas. Furthermore, income level, racism, ethnicity, lifestyle, and insurance status were pointed out as factors related to the death of pancreatic cancer patients in earlier studies [29, 30, 32–34]. However, recent studies have focused on regional disparities [14, 29, 30]. Furthermore, In the case of pancreatic cancer, the survival rate after treatment (surgery or chemotherapy) is high, but in actual healthcare vulnerable areas, surgical treatment is comparatively less than that in non-vulnerable areas [4, 5]. This has been proven in our study as well.

The regional disparity leading to the mortality among pancreatic cancer patients is complex but can be explained by a few mechanisms. Lack of medical resources and low medical accessibility in healthcare vulnerable areas may be the key reason for the delay in diagnosis that renders surgical treatment or chemotherapy unfeasible [14, 30]. In healthcare vulnerable areas, the availability of surgical specialties and centers is low, and pancreatic surgery is technically difficult. Moreover, a surgeon who has not received specialized training in pancreatic cancer surgery may less likely suggest resection [30, 35]. In Korea, most tertiary hospitals are concentrated in the metropolitan area, equipped with various professional manpower, high-quality radiation treatment facilities, and high-quality medical services [36]. However, the lack of medical resources in healthcare vulnerable areas lowers the early diagnosis rate of pancreatic cancer among patients living in such areas, resulting in their lower likelihood of receiving treatments such as surgery or chemotherapy [14]. In fact, in the case of pancreatic cancer, interventions, such as resection, is common during early diagnosis, and in the case of delayed diagnosis, only chemotherapy is deemed suitable [37]. Thus, delay in surgical treatment for cancer due to late screening affects mortality [38]. According to that reported in several previous studies, the diagnosis of pancreatic cancer patients in rural areas is usually possible at an advanced stage [14, 39]. This lack of medical resources may explain why patients with pancreatic cancer living in healthcare vulnerable areas progress further in cancer stages.

Over the last 20 years, Korea has strengthened medical services and accessibility in vulnerable areas through policies such as the Basic Health and Welfare Plan for Rural Areas and Welfare and the establishment of Regional Local Accountable Care hospitals to narrow health imbalances between regions such as access to medical care and equal distribution of medical resources [40, 41]. Despite improvements, our findings suggest that regional health disparities remain a potential obstacle. In particular, challenges, such as access to medical care and lack of resources between regions, can delay diagnosis and increase the risk of death [38]. Thus, policymakers should ensure that health care resources are more evenly distributed across regions considering the accessibility of patients living in particularly vulnerable areas. Moreover, health disparities between regions should be constantly considered through routine assessment of health outcomes between regions.

In our study, mortality was higher in patients with pancreatic cancer in vulnerable areas than those in non-vulnerable areas, especially the untreated group, males, and low-income groups. Even within the untreated group, the difference in mortality rates between healthcare vulnerable areas and non-vulnerable areas may be the result of differences in health behavior as well as differences in medical resources [32]. In patients with pancreatic cancer, unhealthy behaviors such as smoking, drinking alcohol, and obesity are strongly associated with poor health outcomes [33, 42]. In Korea, rural residents were more likely to smoke or be physically active than urban residents, and we can interpret our results through these preceding studies [43] Furthermore, even in Korean male pancreatic cancer patients, the difference in mortality between healthcare vulnerable areas and non-vulnerable areas can be interpreted as a result of differences in health behavior such as smoking and drinking [44]. Finally, within the low-income group of pancreatic cancer patients, the difference in mortality rates between healthcare vulnerable areas and non-vulnerable areas likely the result of health care resources. In previous studies, with similar low income backgrounds, patients who receiving chemotherapy or radiation treatment without surgery were residing more predominantly in the Seoul metropolitan area, where palliative care centers are concentrated [45]. This indicates that even within the low-income group, residence in healthcare vulnerable areas would affect the mortality rate [46].

Our study has several advantages over previous studies. First, we did not categorize the healthcare vulnerable areas into urban or rural areas but, rather applied the PARC index, which used 137 indicators in five areas (medical demand, supply, access, use, and health outcomes) [47]. Thus, the classification of healthcare vulnerable areas is more accurate than in previous studies. Second, to the best of our knowledge, our study is the first in Korea to examine the mortality rate and treatment availability of pancreatic cancer patients according to regional differences using the national data. Previous studies have examined the relationship between mortality using the difference between cancer diagnosis to treatment in lung or gastric cancer patients, but these studies did not consider the regional disparity [38, 48]. Although the use of claims data is limited, we used country cohort data representing the general population of Korea. Therefore, the results of this study can be generalized to the Korean individuals or the entire population of other countries with similar demographic characteristics and can provide a background to alleviate regional disparities in pancreatic cancer patients.

Nevertheless, this study has certain limitations. First, the data on cancer staging could not be adjusted owing to data limitations. To overcome these obstacles and enhance the homogeneity of the study population, the study population was selected to include only patients with initially diagnosed pancreatic cancer or patients who had not undergone previous pancreatic cancer-related surgeries or procedures. Treatment types that could indirectly reflect staging were also separately included in the analysis. However, the effect of other factors affecting treatment cannot be eliminated; hence, additional robust studies are needed to elucidate these associations. Second, because this study used billing data, we could not incorporate several potential covariates into the analysis, including education level, household size and health literacy rate, and smoking and alcohol consumption, which could have an impact on mortality in patients with pancreatic cancer. Therefore, the potential presence of residual confounding factors cannot be completely excluded. Furthermore, regardless of whether pancreatic cancer has occurred or not, living in a healthcare vulnerable area itself may have a low survival rate. Therefore, care should be taken in interpreting the results. Nevertheless, we have incorporated relevant demographic and health-related factors, including disability status, comorbidities, and treatment types, to overcome any of these limitations. Finally, our study could not elucidate clear mechanisms that might support regional differences in treatment or mortality in pancreatic cancer patients. Therefore, in the future, it is necessary to derive robust associations of factors for regional disparities in their relationship.

## Conclusions

In conclusion, this study identified the regional disparity between mortality and treatment after cancer diagnosis by dividing pancreatic cancer patients according to their residence in healthcare vulnerable and non-vulnerable areas using the PARC index in a large, nationally representative sample. According to the results of this study, patients with pancreatic cancer in healthcare vulnerable areas were less likely to receive treatment (especially chemotherapy) compared with patients in non-vulnerable areas, and the mortality rate was also higher in such patients. This may be a result of the delayed diagnosis. Therefore, the results of this study highlight the need for further studies to identify inter-regional factors related to the treatment and survival of pancreatic cancer patients.

## Supplementary Information

**Additional file 1. Supplementary Table 1.** List of surgeries and chemotherapies included in this study.

**Additional file 2. Supplementary Table 2. **General characteristics of the study population according to the treatment of pancreatic cancer. 

**Additional file 3. Supplementary Table 3. **General characteristics of the study population according to the pancreatic cancer mortality.

## Data Availability

Data are available on reasonable request. The data can be accessed on the National Health Insurance Data Sharing Service homepage of the National Health Insurance Service (http://nhiss.nhis.or.kr). Applications to use the NHIS data will be reviewed by the inquiry committee of research support and, once approved, raw data will be provided to the applicant for a fee. Contact information for a data access committee is listed as follows: National Health Insurance Sharing Service, Tel: 82–33–736–2432; Official internet site: https://nhiss.nhis.or.kr/bd/ay/bdaya001iv.do

## Abbreviations

PARC: Position value for Relatively Active Company index
NHIS-NSC: Korea National Health Insurance Service-National Sample Cohort
CCI: Charlson Comorbidity Index
ICD: International Classification of Diseases
HR: Hazard Ratios
CI: Confidence Intervals
OR: Odds ratio
