# Supplementary material for: Healthcare vulnerability disparities in pancreatic cancer treatment and mortality using the Korean National Sample Cohort: a retrospective cohort study
Source: BMC Cancer. 2022 Aug 27;22:925. doi: 10.1186/s12885-022-10027-2 (PMC9419365; doi:10.1186/s12885-022-10027-2)
Supplement: Supplementary file 1 — Additional file 1. Supplementary Table 1. List of surgeries and chemotherapies included in this study. [file 12885_2022_10027_MOESM1_ESM.docx]

**Supplementary Table 1.** List of surgeries and chemotherapies included in this study

| **Chemotherapy** | |
| --- | --- |
| **Procedure Code** | **Medicine** |
| 164901BIJ  164930BIJ | Gemcitabine HCl 1000 mg |
| 164902BIJ  164931BIJ | Gemcitabine HCl 200 mg |
| 164903BIJ  164932BIJ | Gemcitabine HCl 2280 mg |
| 161401BIJ  161430BIJ | Fluorouracil 250 mg |
| 161402BIJ  503701BIJ | Fluorouracil 500 mg |
| **Surgical surgery** | |
| **Procedure Code** | **Type of surgery** |
| Q7565 | Distal pancreatectomy |
| Q7567 | Spleen-preserving distal pancreatectomy |
| Q7563 | Subtotal pancreatectomy |
| Q7571 | Pancreaticoduodenectomy (Whipple’s operation) |
| Q7572 | Pancreaticoduodenectomy (Pylorus-preserving operation) |
| Q7562 | Duodenum-preserving pancreatic head resection |
| Q7561 | Total pancreatectomy |
| QZ964 | Revo-i central pancreatectomy |
| QZ961 | da Vinci central pancreatectomy |
