# Supplementary material for: Healthcare vulnerability disparities in pancreatic cancer treatment and mortality using the Korean National Sample Cohort: a retrospective cohort study
Source: BMC Cancer. 2022 Aug 27;22:925. doi: 10.1186/s12885-022-10027-2 (PMC9419365; doi:10.1186/s12885-022-10027-2)
Supplement: Supplementary file 2 — Additional file 2. Supplementary Table 2. General characteristics of the study population according to the treatment of pancreatic cancer. [file 12885_2022_10027_MOESM2_ESM.docx]

Supplementary Table 2. General characteristics of the study population according to the treatment of pancreatic cancer

| **Variables** | **Pancreatic Cancer Treatment** | | | | | | | | | |
| --- | --- | --- | --- | --- | --- | --- | --- | --- | --- | --- |
|  | **Total** | |  | **Yes** | | | | **No** | | ***P*-value** |
|  |  |  |  | **Surgery** | | **Chemotherapy** | |  |  |  |
|  | **N** | **%** |  | **N** | **%** | **N** | **%** | **N** | **%** |  |
| **Total** | 1,975 | 100.0 |  | 313 | 15.8 | 547 | 27.7 | 1,115 | 56.46 |  |
| **Healthcare Vulnerability** |  |  |  |  |  |  |  |  |  | 0.0006 |
| Vulnerable region | 279 | 14.1 |  | 35 | 12.5 | 57 | 20.4 | 187 | 67.0 |  |
| Non-vulnerable region | 1,696 | 85.9 |  | 278 | 16.4 | 490 | 28.9 | 928 | 54.7 |  |
| **Sex** |  |  |  |  |  |  |  |  |  | 0.0995 |
| Male | 1,093 | 55.3 |  | 176 | 16.1 | 322 | 29.5 | 595 | 54.4 |  |
| Female | 882 | 44.7 |  | 137 | 15.5 | 225 | 25.5 | 520 | 59.0 |  |
| **Age (years)** |  |  |  |  |  |  |  |  |  | < 0.0001 |
| <50 | 173 | 8.8 |  | 39 | 22.5 | 50 | 28.9 | 84 | 48.6 |  |
| 50-60 | 311 | 15.7 |  | 60 | 19.3 | 122 | 39.2 | 129 | 41.5 |  |
| 60-70 | 503 | 25.5 |  | 105 | 20.9 | 187 | 37.2 | 211 | 41.9 |  |
| 70-80 | 598 | 30.3 |  | 88 | 14.7 | 165 | 27.6 | 345 | 57.7 |  |
| ≥80 | 390 | 19.7 |  | 21 | 5.4 | 23 | 5.9 | 346 | 88.7 |  |
| **Household income** |  |  |  |  |  |  |  |  |  | 0.7298 |
| Low | 437 | 22.1 |  | 62 | 14.2 | 128 | 29.3 | 247 | 56.5 |  |
| Mid-low | 621 | 31.4 |  | 106 | 17.1 | 165 | 26.6 | 350 | 56.4 |  |
| Mid-high | 917 | 46.4 |  | 145 | 15.8 | 254 | 27.7 | 518 | 56.5 |  |
| **Medical Insurance** |  |  |  |  |  |  |  |  |  | 0.5724 |
| Insurance Coverage (Regional) | 639 | 32.4 |  | 94 | 14.7 | 174 | 27.2 | 371 | 58.1 |  |
| Insurance Coverage (corporate) | 1,279 | 64.8 |  | 213 | 16.7 | 357 | 27.9 | 709 | 55.4 |  |
| Medical Aid | 57 | 2.9 |  | 6 | 10.5 | 16 | 28.1 | 35 | 61.4 |  |
| **Disorder** |  |  |  |  |  |  |  |  |  | 0.0014 |
| No | 1,828 | 92.6 |  | 293 | 16.0 | 523 | 28.6 | 1,012 | 55.4 |  |
| Yes | 147 | 7.4 |  | 20 | 13.6 | 24 | 16.3 | 103 | 70.1 |  |
| **CCI** |  |  |  |  |  |  |  |  |  | < 0.0001 |
| 0 | 353 | 17.9 |  | 57 | 16.1 | 113 | 32.0 | 183 | 51.8 |  |
| 1-2 | 885 | 44.8 |  | 172 | 19.4 | 280 | 31.6 | 433 | 48.9 |  |
| ≥3 | 737 | 37.3 |  | 84 | 11.4 | 154 | 20.9 | 499 | 67.7 |  |

CCI, Charlson Comorbidity Index
