# Supplementary material for: Healthcare vulnerability disparities in pancreatic cancer treatment and mortality using the Korean National Sample Cohort: a retrospective cohort study
Source: BMC Cancer. 2022 Aug 27;22:925. doi: 10.1186/s12885-022-10027-2 (PMC9419365; doi:10.1186/s12885-022-10027-2)
Supplement: Supplementary file 3 — Additional file 3. Supplementary Table 3. General characteristics of the study population according to the pancreatic cancer mortality. [file 12885_2022_10027_MOESM3_ESM.docx]

Supplementary Table 3. General characteristics of the study population according to the pancreatic cancer mortality

| **Variables** | **Total** | | **3-month mortality** | | | | ***P*-value** | **6-month mortality** | | | | ***P*-value** | **1-year Mortality** | | | | ***P*-value** |
| --- | --- | --- | --- | --- | --- | --- | --- | --- | --- | --- | --- | --- | --- | --- | --- | --- | --- |
|  |  |  | **No** | | **Yes** | |  | **No** | | **Yes** | |  | **No** | | **Yes** | |  |
| **Total** | 1,975 | (100.0) | 1,593 | (80.7) | 382 | (19.3) |  | 1,350 | (68.4) | 625 | (31.6) |  | 1,080 | (54.7) | 895 | (45.3) |  |
| **Healthcare Vulnerability** |  |  |  |  |  |  | 0.0010 |  |  |  |  | 0.0139 |  |  |  |  | 0.0587 |
| Vulnerable region | 279 | (14.1) | 205 | (73.5) | 74 | (26.5) |  | 173 | (62.0) | 106 | (38.0) |  | 138 | (49.5) | 141 | (50.5) |  |
| Non-vulnerable region | 1,696 | (85.9) | 1,388 | (81.8) | 308 | (18.2) |  | 1,177 | (69.4) | 519 | (30.6) |  | 942 | (55.5) | 754 | (44.5) |  |
| **Pancreatic Cancer Treatment** |  |  |  |  |  |  | <.0001 |  |  |  |  | <.0001 |  |  |  |  | <.0001 |
| Yes | 860 | (43.5) | 808 | (94.0) | 52 | (6.0) |  | 714 | (83.0) | 146 | (17.0) |  | 535 | (62.2) | 325 | (37.8) |  |
| No | 1,115 | (56.5) | 785 | (70.4) | 330 | (29.6) |  | 636 | (57.0) | 479 | (43.0) |  | 545 | (48.9) | 570 | (51.1) |  |
| **Sex** |  |  |  |  |  |  | 0.7828 |  |  |  |  | 0.3872 |  |  |  |  | 0.5063 |
| Male | 1,093 | (55.3) | 884 | (80.9) | 209 | (19.1) |  | 756 | (69.2) | 337 | (30.8) |  | 605 | (55.4) | 488 | (44.6) |  |
| Female | 882 | (44.7) | 709 | (80.4) | 173 | (19.6) |  | 594 | (67.3) | 288 | (32.7) |  | 475 | (53.9) | 407 | (46.1) |  |
| **Age** |  |  |  |  |  |  | <.0001 |  |  |  |  | <.0001 |  |  |  |  | <.0001 |
| <50 | 173 | (8.8) | 162 | (93.6) | 11 | (6.4) |  | 149 | (86.1) | 24 | (13.9) |  | 138 | (79.8) | 35 | (20.2) |  |
| 50-60 | 311 | (15.7) | 284 | (91.3) | 27 | (8.7) |  | 251 | (80.7) | 60 | (19.3) |  | 195 | (62.7) | 116 | (37.3) |  |
| 60-70 | 503 | (25.5) | 436 | (86.7) | 67 | (13.3) |  | 388 | (77.1) | 115 | (22.9) |  | 317 | (63.0) | 186 | (37.0) |  |
| 70-80 | 598 | (30.3) | 471 | (78.8) | 127 | (21.2) |  | 379 | (63.4) | 219 | (36.6) |  | 298 | (49.8) | 300 | (50.2) |  |
| ≥80 | 390 | (19.7) | 240 | (61.5) | 150 | (38.5) |  | 183 | (46.9) | 207 | (53.1) |  | 132 | (33.8) | 258 | (66.2) |  |
| **Household income** |  |  |  |  |  |  | 0.7922 |  |  |  |  | 0.5266 |  |  |  |  | 0.1328 |
| Low | 437 | (22.1) | 349 | (79.9) | 88 | (20.1) |  | 289 | (66.1) | 148 | (33.9) |  | 222 | (50.8) | 215 | (49.2) |  |
| Mid-low | 621 | (31.4) | 506 | (81.5) | 115 | (18.5) |  | 428 | (68.9) | 193 | (31.1) |  | 354 | (57.0) | 267 | (43.0) |  |
| Mid-high | 917 | (46.4) | 738 | (80.5) | 179 | (19.5) |  | 633 | (69.0) | 284 | (31.0) |  | 504 | (55.0) | 413 | (45.0) |  |
| **Medical Insurance** |  |  |  |  |  |  | 0.9350 |  |  |  |  | 0.5069 |  |  |  |  | 0.3775 |
| Insurance Coverage(Regional) | 639 | (32.4) | 517 | (80.9) | 122 | (19.1) |  | 436 | (68.2) | 203 | (31.8) |  | 351 | (54.9) | 288 | (45.1) |  |
| Insurance Coverage(corporate) | 1,279 | (64.8) | 1,031 | (80.6) | 248 | (19.4) |  | 879 | (68.7) | 400 | (31.3) |  | 703 | (55.0) | 576 | (45.0) |  |
| Medical Aid | 57 | (2.9) | 45 | (78.9) | 12 | (21.1) |  | 35 | (61.4) | 22 | (38.6) |  | 26 | (45.6) | 31 | (54.4) |  |
| **Disorder** |  |  |  |  |  |  | 0.0025 |  |  |  |  | <.0001 |  |  |  |  | 0.0013 |
| No | 1,725 | (87.3) | 1,409 | (81.7) | 316 | (18.3) |  | 1,207 | (70.0) | 518 | (30.0) |  | 967 | (56.1) | 758 | (43.9) |  |
| Yes | 250 | (12.7) | 184 | (73.6) | 66 | (26.4) |  | 143 | (57.2) | 107 | (42.8) |  | 113 | (45.2) | 137 | (54.8) |  |
| **CCI** |  |  |  |  |  |  | 0.0973 |  |  |  |  | 0.2826 |  |  |  |  | 0.0001 |
| 0 | 353 | (17.9) | 272 | (77.1) | 81 | (22.9) |  | 232 | (65.7) | 121 | (34.3) |  | 179 | (50.7) | 174 | (49.3) |  |
| 1-2 | 885 | (44.8) | 729 | (82.4) | 156 | (17.6) |  | 600 | (67.8) | 285 | (32.2) |  | 453 | (51.2) | 432 | (48.8) |  |
| ≥3 | 737 | (37.3) | 592 | (80.3) | 145 | (19.7) |  | 518 | (70.3) | 219 | (29.7) |  | 448 | (60.8) | 289 | (39.2) |  |
